# Chest wall resection and reconstruction using titanium micromesh covered with Marlex mesh for metastatic follicular thyroid carcinoma: a case report

**DOI:** 10.4076/1752-1947-3-7259

**Published:** 2009-06-08

**Authors:** Nobuyasu Suganuma, Nobuyuki Wada, Hiromasa Arai, Hirotaka Nakayama, Keita Fujii, Katsuhiko Masudo, Norio Yukawa, Yasushi Rino, Munetaka Masuda, Toshio Imada

**Affiliations:** 1 Department of Surgery, Yokohama City University Hospital and Medical Center, Fukuura, Kanazawa, Yokohama, Kanagawa, Japan

## Abstract

**Introduction:**

The distant metastases from differentiated thyroid carcinomas are often untreatable. In particular, bone metastasis is significantly related to poor prognosis since radioactive iodine therapy is generally less effective. Therefore, surgical resection is considered one of the treatments for patients with bone metastases. We report chest wall resection and reconstruction using titanium micromesh covered with polypropylene mesh (Marlex mesh) for metastatic rib bones as a result of follicular thyroid carcinoma.

**Case presentation:**

A 51-year-old man was referred to our institution with a painful chest wall tumor. He presented with a 15 × 10 cm bony swelling on the left chest wall and multiple small lung nodules from follicular thyroid carcinoma. Completion total thyroidectomy, chest wall resection and reconstruction using titanium micromesh covered with Marlex mesh were performed. There were no critical complications associated with surgical treatments and tumor pain disappeared during the postoperative period. Then, he received radioactive iodine therapy and the uptake of radioactive iodine was well observed in bilateral lung fields.

**Conclusion:**

Reconstruction using titanium micromesh covered with Marlex mesh is possible for repairing the wide chest wall resection required for thyroid carcinoma metastasis. This technique would help to enhance treatment efficacy in the combination therapy of radioactive iodine and surgery in patients with large thyroid carcinoma metastasis in the chest wall.

## Introduction

Differentiated thyroid carcinomas (DTC) are usually curable, but the distant metastases are often untreatable. Lungs and bones are the major metastatic sites for DTC and radioactive iodine (RI) therapy is important in the treatment of such distant metastases. RI therapy is generally less effective in bone metastasis due to the low uptake of RI [1] and the occurrence of bone metastases is significantly related to poor prognosis [2,3]. Therefore, if possible, surgical resection should be considered one of the treatments of choice for patients with bone metastases. Solitary sternal metastasis can be a good indicator for surgical resection and reconstruction and these treatments have been successfully performed in clinical practice [4-6]. In addition, elimination of large bone metastases is considered to facilitate other distant metastases, mainly lung metastases, to uptake RI effectively. Such an enhancement effect is expected in RI treatment for DTC patients with multiple metastases. Thus, surgical resection of bone metastases contributes to the efficacy of RI therapy and is associated with favorable prognosis and improved quality-of-life (QOL) [7].

To our knowledge, this is the first report of chest wall resection and reconstruction using titanium micromesh covered with polypropylene mesh (Marlex mesh) for metastatic rib bones as a result of follicular thyroid carcinoma (FTC). There were no critical complications such as flail chest, dyspnea, or infection. Chest wall pain as the chief complaint disappeared after surgery and this procedure improved the patient's QOL.

## Case presentation

**Figure 1:**
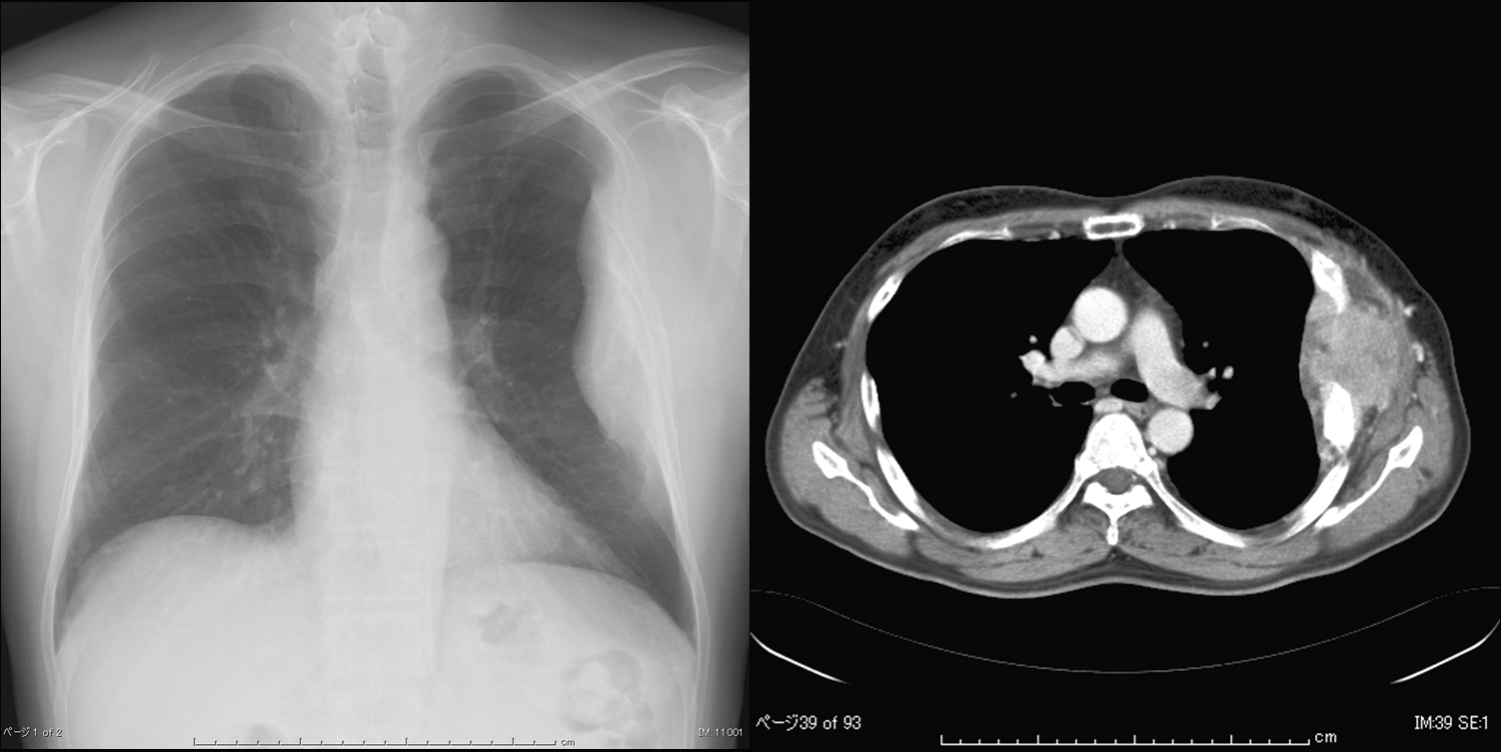
**Chest X-ray and computed tomography scan before surgery.** Chest X-ray and computed tomography scan showed a 14 × 8 cm tumor of the left chest wall in the left ribs.

**Figure 2:**
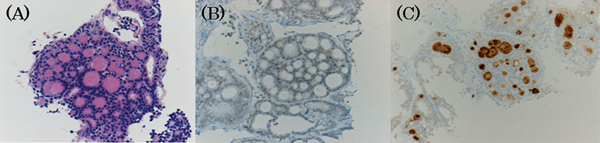
**Core-needle biopsy of the left chest wall tumor.** Hematoxylin and eosin stain **(A)** revealed follicular growth, and immunohistochemical stain was positive for thyroid transcription factor-1 **(B)** and thyroglobulin **(C)**.

A 51-year-old man, who had a treatment history for thyroid tumor elsewhere, was referred to our institution in June 2006 due to a painful chest wall tumor. He had undergone right hemi-thyroidectomy as a 42-year-old but the detailed information was not available at presentation in our institution. On physical examination, the patient presented with a 15 × 10 cm bony swelling on the left chest wall. The thyroid tumor was not palpable in the residual left lobe of the thyroid gland. Ultrasound (US) examination subsequently revealed no thyroid tumor and no regional lymphadenopathy. Laboratory data showed an extremely elevated level of serum thyroglobulin (Tg) (12,000 ng/mL) with normal thyroid function. Chest X-ray and computed tomography (CT) scan showed a 14 × 8 cm tumor in the left 3rd to 6th ribs and multiple small lung nodules (Figure 1). Core-needle biopsy was performed on the left chest wall tumor. Hematoxylin-eosin staining (Figure 2A) revealed follicular growth in the tumor specimen obtained and immunohistochemical staining was positive for Tg (Figure 2B) and thyroid transcription factor-1 (Figure 2C). Thus, we diagnosed that the previously resected thyroid tumor was FTC and his painful chest wall tumor and multiple lung nodules were metachronous distant metastases from the previously resected FTC.

**Figure 3:**
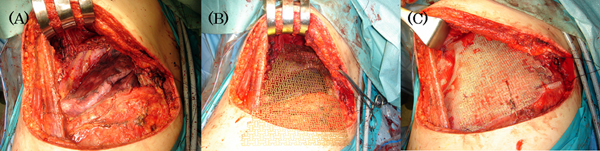
**Reconstruction of the left chest wall defect.(A)** The left chest wall tumor was resected with the 3rd to 6th ribs. **(B)** Titanium micromesh and Marlex mesh were appropriately cut larger than the skeletal defect. **(C)** Titanium micromesh covered with Marlex mesh was sutured to the 2nd to 8th ribs with titanium wires to improve rigidity.

Complete total thyroidectomy and chest wall resection and reconstruction using titanium micromesh covered with Marlex mesh were performed in August 2006. First, complete total thyroidectomy of the residual left thyroid gland was performed in the supine position before chest wall resection. There was no thyroid tumor in the resected lobe, as diagnosed pre-operatively. Then, the patient position was changed to the right lateral position. First, we observed the chest wall tumor from inside the thoracic cavity with a thoracoscope, endoscope technique, to determine the marginal line for resection. Next, a post-lateral incision was made. The anterior serratus muscles, 3rd to 6th ribs, and those intercostal muscles with 2 cm margins on both sides were resected (Figure 3A). The thoracodorsal muscles were not resected because there was no invasion. After the resection, the defect was approximately 20 × 15 cm in size. Finally, we reconstructed the chest wall defect using titanium micromesh covered with Marlex mesh, with a sandwich method. Pieces of Marlex mesh and titanium micromesh were appropriately cut to be larger than the skeletal defect and the titanium micromesh was sandwiched between two Marlex meshes (Figure 3B). Then, we sutured the mesh to the 2nd and 8th ribs and the dorsal edges of the 3rd to 6th ribs with titanium wires to provide rigidity (Figure 3C). Two drainage tubes were inserted into the left thoracic cavity and the chest wall was suitably closed. The whole surgical procedure including total thyroidectomy and resection and reconstruction of the chest wall was accomplished in 10.7 hours of operation time and 1050 mL of blood loss was recorded during the operation The patient did not receive a blood transfusion.

**Figure 4:**
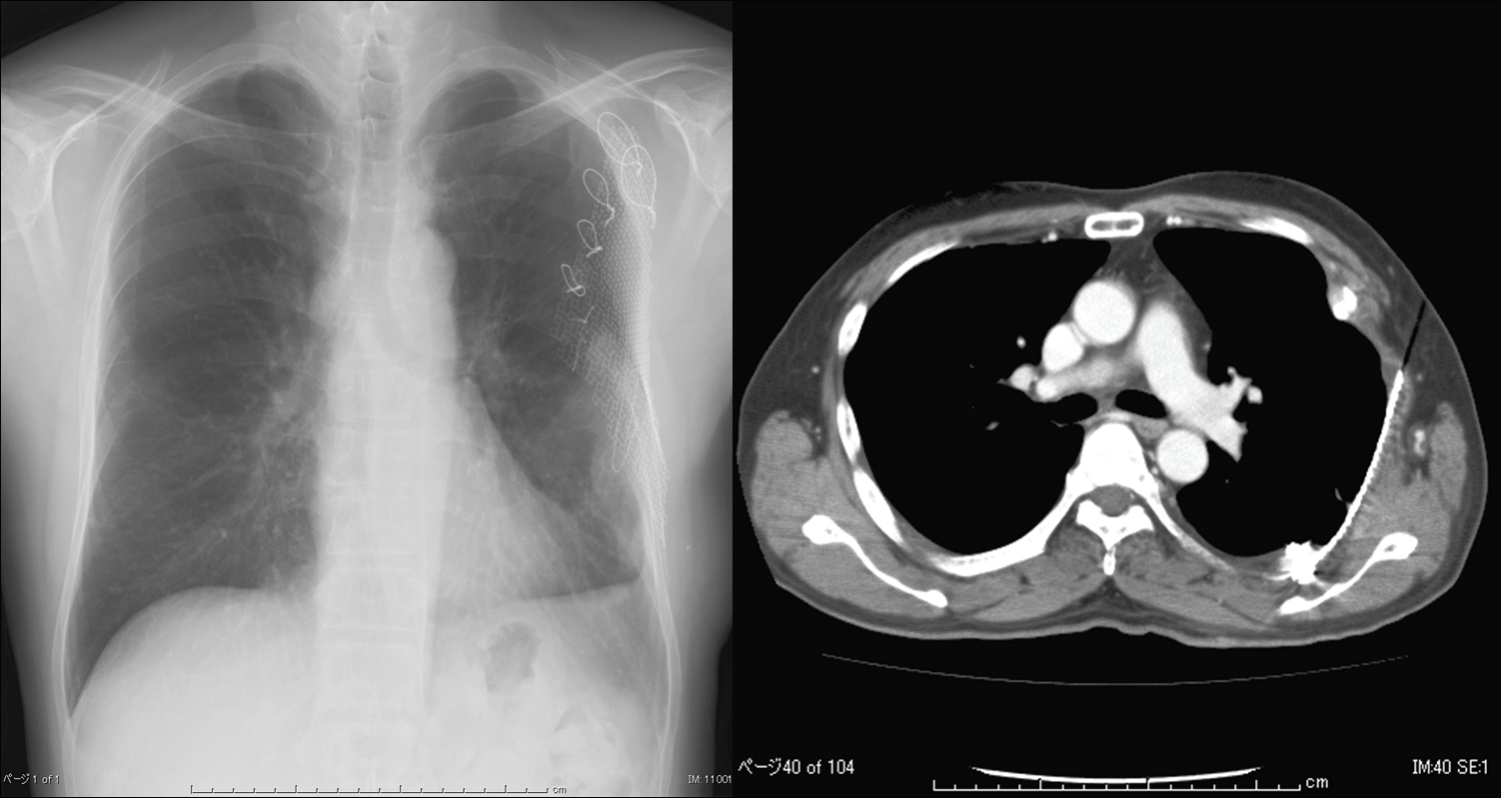
**Chest X-ray and computed tomography scan after surgery.** Chest X-ray and computed tomography scan showed that the left lung had completely expanded and reconstruction of the thoracic defect was successfully achieved.

There were no critical complications associated with the surgical treatments. Components of flail chest, dyspnea, and surgical site infection were not observed. Tumor pain disappeared during the postoperative period. Chest X-ray and CT scan showed that the left lung had completely expanded and reconstruction of the thoracic defect was successfully achieved (Figure 4). Respiratory function was approximately equivalent before and after surgery. Histopathological examination revealed that the resected specimens were metastatic lesions of poorly differentiated FTC. The patient was discharged on the 11th postoperative day. The level of serum Tg decreased to 2800 ng/mL 2 months after the resection. He received RI ablation (50 mCi) and uptake was observed in the thyroid bed in November 2006, 3 months after surgery. Unfortunately, in December 2006, he developed spinal bone metastases and this caused spinal compression and paralysis at the level of the 7th thoracic vertebrae. Orthopedic surgery was immediately performed to fix the spinal aliments with rod instruments and paralysis was dramatically improved. Then, he received a therapeutic dose of RI (100 mCi) in May 2007, 9 months after our surgery. Uptake of RI was well observed in bilateral lung fields. Although the RI treatment was considered effective for lung metastases, spinal bone metastasis gradually progressed. Paraplegia and bladder bowel disturbance were present in August 2007, 12 months after our surgery, and his general condition deteriorated day by day. Eventually, he died of disease in March 2008 19 months after our surgery due to his reduced general condition. This patient never suffered painful chest wall tumor after the resection and lung metastasis was clinically controlled during the follow-up period.

## Discussion

Distant metastases,mainly in the lung and bone, occur in 10% to 20% of patients with DTC. RI therapy is essential to treat such metastatic disease. Lung metastases usually respond to RI treatment, however bone metastases uncommonly respond to RI therapy and are associated with poor prognosis [1-3,8]. Casara *et al.*[3] reported that less than 5% of patients with bone metastases achieve complete remission despite the fact that RI uptake was observed in 60% of patients, whereas more than 35% of patients with lung metastases were considered to achieve complete remission after RI therapy. The 10-year survival rates for patients with bone and lung metastases were 15% and 53%, respectively. Because of the low remission rate in RI therapy and poor prognosis in patients with bone metastases, the surgical approach should be considered as one of the treatments of choice for bone metastasis, if possible. Curative resection of solitary bone metastasis is associated with improved survival, especially in younger patients [7,9-11]. Moreover, patients' QOL is often improved by surgical removal of the metastases due to the rapid relief of symptoms. Thus, surgical resection of bone metastases is considered valuable. In addition, external beam radiotherapy or embolization has been reported to be effective as palliative therapy for unresectable or multiple bone metastases [12].

Our patient had large bone metastases at the left chest wall and multiple small lung nodules. We considered that it was not enough to treat these advanced lesions with RI therapy alone because of the decreased effectiveness of RI therapy to large bone metastases and the decreased RI delivery to lung metastases. Therefore, we performed chest wall resection followed by reconstruction. Removal of large bone metastases was expected to facilitate other distant metastases to uptake RI efficiently. In fact, RI was well concentrated to lung metastases after surgery. Furthermore, this patient became free from left chest wall pain with limited analgesics after surgery. Thus, such surgical treatment is considered to contribute to both the efficacy of RI uptake and better patients' QOL, although just in restricted cases.

There are some reports on the surgical methods used for reconstruction after sternal resection [4-6]. Marlex mesh is reported to be easy to handle, has a high affinity for tissues and is resistant to infections, but it may have a paradoxical chest wall motion because of the lack of rigidity when the defect is large. On the other hand, a metal plate can retain the rigidity and prevent a flail chest, but its affinity to tissue is insufficient and lung injury may occur. Briccoli *et al.*[13] reported that sternal reconstruction with Marlex mesh and a titanium plate after sternotomy resulted in satisfactory surgical treatment without a flail chest. We used a titanium micromesh, which has many holes on a titanium plate, moderate malleability and sufficient rigidity, and covered with Marlex mesh for reconstruction of the wide defect after thoracic wall resection. This procedure showed good affinity to tissues, prevention of paradoxical respiration and an acceptable level of radiolucency. To our knowledge, this is the first report using titanium micromesh covered with Marlex mesh for reconstruction after the resection of chest wall metastases from thyroid carcinoma.

## Conclusion

Reconstruction using a titanium micromesh covered with Marlex mesh is possible for repairing the wide chest wall resection from thyroid carcinoma metastasis. This technique could help to enhance the treatment efficacy in the combination therapy of RI and surgery in patients with large thyroid carcinoma metastases in the chest wall.

## Consent

Written informed consent was obtained from the patient for publication of this case report and any accompanying images. A copy of the written consent is available for review by the Editor-in-Chief of this journal.

## Competing interests

The authors declare that they have no competing interests.

## Authors' contributions

HA, HN and KF participated in the collection of data and patient care. KM and NY were involved in the preparation of the manuscript. NW provided collection of data, patient care and editing of the manuscript. YR, MM and TI participated in final revision of the manuscript and guidance. All authors read and approved the final manuscript.
